# 亲和超滤色谱-质谱技术在天然活性物质筛选中的研究进展

**DOI:** 10.3724/SP.J.1123.2025.08020

**Published:** 2026-08-08

**Authors:** Yongbing XU, Yujia NIU, Mingquan GUO

**Affiliations:** 1. 中国科学院宁波材料技术与工程研究所，先进诊疗材料与技术实验室，浙江 宁波 315201; 1. Laboratory of Advanced Theranostic Materials and Technology，Ningbo Institute of Materials Technology and Engineering，Chinese Academy of Sciences，Ningbo 315201，China; 2. 宁波慈溪生物医学工程研究所，浙江 宁波 315300; 2. Ningbo Cixi Institute of Biomedical Engineering，Ningbo 315300，China; 3. 浙江工业大学药学院，浙江 杭州 310014; 3. School of Pharmacy，Zhejiang University of Technology，Hangzhou 310014，China

**Keywords:** 天然活性物质, 亲和超滤, 液相色谱-质谱法, 多靶点, 活性成分筛选, natural active substances, affinity ultrafiltration （AUF）, liquid chromatography-mass spectrometry （LC-MS）, multi-targets, active component screening

## Abstract

药用植物（包括中草药及其复方）是天然活性化合物的丰富来源，这些天然活性成分在人类健康的维护和疾病防治方面发挥着重要作用。然而，药用植物及中草药中的化学成分众多且十分复杂，如何快速筛选并阐释其中的主要活性物质一直是其药效物质基础研究以及新药研发的难题。亲和超滤-液相色谱-质谱（AUF-LC-MS）技术是一种将亲和捕获、超滤分离并结合色谱-质谱联用分析的快速筛选和鉴定天然活性小分子物质的重要方法，具有所需样品少、特异性强、灵敏度高、快速高效、操作简便等诸多优点。该技术通过利用药用植物或中草药提取物与靶标之间的亲和力差异，从复杂的基质中快速筛选并鉴定出活性成分，特别适用于药用植物及中药等复杂提取物。本研究综述了AUF-LC-MS技术的原理、特点以及该技术近10年（2015-2025）在天然活性成分筛选中的应用进展，包括对不同水平的AUF-LC-MS筛选（化合物水平、中药单方水平和中药复方水平）进行了归纳和总结。另外，依据靶点数量的不同，将中药单方水平的AUF-LC-MS筛选分类为单靶点筛选、双靶点筛选和多靶点筛选进一步细化并总结。同时，对该技术与其他不同的天然活性物质筛选方法进行了比较，并对该技术的未来研究方向进行了展望，以期为中药药效物质基础研究以及新药研发提供新的思路。

## Abstract (en)

Medicinal plants serve as a valuable source of naturally occurring bioactive compounds， which have played a vital role in promoting human health， preventing and treating various diseases for thousands of years. However， the phytochemical profiles of medicinal plants， including traditional Chinese herbal medicines （TCHMs）， are notably complex and highly diverse， posing a tough challenge for the rapid screening and identification of their potential bioactive constituents. This challenge is central to pharmacological research and drug development efforts in medicinal plants including TCHMs. Therefore， the rapid and efficient screening of bioactive components from complex natural products has become a key research direction in academia. To address this issue， affinity ultrafiltration coupled with liquid chromatography-mass spectrometry （AUF-LC-MS） has emerged as an efficient technique that integrates affinity-based capture， ultrafiltration， and liquid chromatographic-mass spectrometric analysis to enable rapid screening and characterization of potential small-molecule bioactive compounds. Known for its low sample consumption， high specificity， exceptional sensitivity， operational simplicity and high efficiency， this approach leverages specific interactions between target molecules and chemical extracts to identify bioactive ligands from complex mixtures， making it particularly suitable for studying intricate matrices such as medicinal plant extracts and TCHM formulations. This study summarizes the principles， characteristics， and recent applications of AUF-LC-MS technology in the screening of potential active components in medicinal plants including TCHMs over the past decade （2015-2025）. Based on different levels of AUF-LC-MS screening， including compound level， single TCHM level， and TCHM formula level， a comprehensive summary is provided. AUF-LC-MS screening of single TCHM level is further analyzed and summarized based on the number of targets， including single target screening， dual-targets screening， and multi-targets screening. At the same time， comparisons were made between other different natural active substance screening approaches and the AUF-LC-MS method. In addition， it outlines prospective research directions for the further development of AUF-LC-MS with the aim of providing new insights for the study of the pharmacological substances of TCHMs and the development of new drugs.

药用植物是天然活性化合物的丰富来源，数千年来，这些天然活性成分在人类健康的维护和疾病防治方面发挥着十分重要的作用^［1］^。据统计，1981~2019年全球批准上市的新药共有1 881种，其中超过50%来源于天然产物或者与天然产物相关^［2］^。然而，药用植物提取物中通常含有成百上千种具有不同生物功能的化学成分，其中具体发挥特定功能的生物活性物质结构与作用机制多数尚未明晰。传统植物化学方法从复杂的药用植物中分离后筛选天然活性化合物常常费力、耗时且昂贵，这严重阻碍了药用植物中天然活性化合物的挖掘步伐。因此，如何从复杂的天然产物中快速高效地筛选活性物质成为学界重要的研究方向之一。

为探索药用植物中的生物活性物质并揭示其潜在药理机制，一些快速高效的筛选研究策略应运而生，例如网络药理学、分子对接、细胞膜色谱、固定化酶技术等^［3，4］^。其中，基于小分子配体与药物靶标特异性结合的亲和超滤液相色谱-质谱技术（affinity ultrafiltration-liquid chromatography-mass spectrometry， AUF-LC-MS），由于具有灵敏度高、选择性强、所需样品少、操作简单等多种优点，已成为当前非常重要的生物活性成分筛选策略之一，并已成功应用于许多天然药物活性物质的筛选中^［5］^，因此非常有必要对这一方法的特点、研究进展和未来发展趋势进行总结和展望。

本文以不同水平的亲和超滤筛选研究为切入点，结合本课题组近年来的研究进展，对AUF-LC-MS技术在快速筛选复杂药用植物中天然活性物质的应用进展进行综述。重点对中药单方水平的AUF-LC-MS研究进展进行了详细总结，包括中药单方水平的单靶点筛选、双靶点筛选和多靶点筛选。同时对当前其他一些不同的天然活性物质筛选方法与AUF-LC-MS方法进行比较，另外对AUF-LC-MS的未来研究方向进行了展望，以期为今后快速靶向筛选复杂药用植物中的活性物质及新药研发提供新的思路。

## 1 AUF-LC-MS简介及其发展历程

亲和超滤色谱-质谱技术是一种将亲和捕获、超滤分离与色谱-质谱技术联用的快速筛选和鉴定生物活性小分子的方法^［6］^，其简要流程示意图如图1所示。其工作原理是将生物靶酶与药用植物提取物进行孵育，药用植物提取物中的活性成分能够与靶酶形成特定的复合物，然后利用超滤膜将未结合的成分与复合物分开，接着利用有机溶剂将活性成分从其与靶酶结合形成的复合物中解离出来，再利用液相色谱-质谱联用技术对这些解离出来的活性化合物进行分析和鉴定，最后再对筛选出来的活性物质进行活性验证。

**图1:**
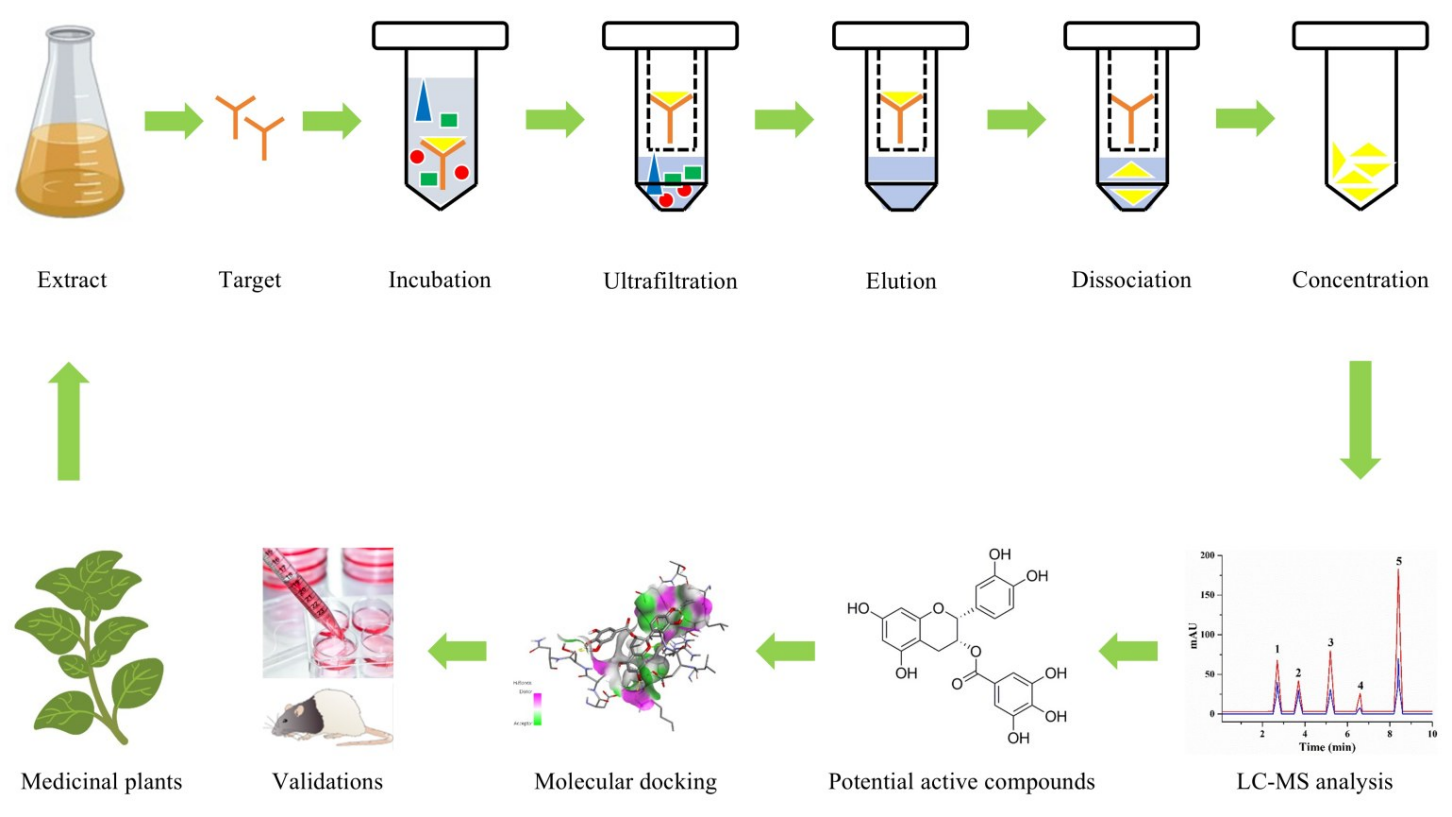
亲和超滤色谱-质谱技术工作原理示意图

亲和超滤主要分为离心超滤（centrifugal ultrafiltration-MS， CU-MS）和脉冲超滤（pulsed ultrafiltration-MS， PU-MS），这两种方法的基本原理相同，均是通过半透膜的选择性分离和靶标的特异性结合达到富集和分离活性小分子配体的目的^［7］^。1997年，Van Breemen等^［8］^首次以血清白蛋白和腺苷脱氨酶为靶标，成功地将AUF-LC-MS技术应用于组合化合物库中活性成分的筛选，使得这一联用技术在复杂体系中快速筛选活性物质的优势逐渐显现，而后越来越多的研究将其运用到天然活性物质的筛选中。

本研究以“亲和超滤”“超滤质谱”“超滤筛选”“affinity ultrafiltration”和“AUF-LC-MS”为关键词，在中国知网、万方、Web of Science和PubMed数据库中进行检索，检索时间跨度为2015年1月1日至2025年6月30日，经剔除不相关及重复文献，共有亲和超滤相关文献312篇，对这312篇文献发表时间的分布特征进行分析，结果如图2所示。近10年来，亲和超滤筛选的文章呈现逐年递增的趋势，仅在2020年有所下降，其可能的原因是2020年新冠疫情导致的实验性科研进展短时间停滞，但在2021年便又恢复了增长的趋势，表明亲和超滤筛选方法正受到越来越多研究者的关注。

**图2:**
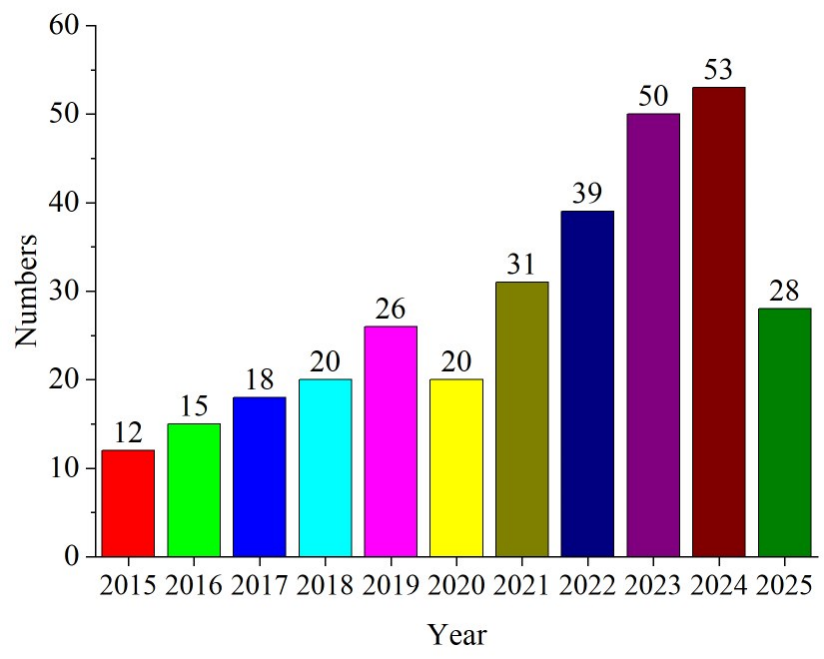
近10年亲和超滤色谱-质谱技术相关文献数量统计图

另外，对这312篇亲和超滤相关文献中所涉及的靶点进行分析和总结，依据靶点所对应的活性可将相关超滤靶点分为降糖、降脂、降尿酸、抗炎、抗肿瘤、抗病毒、抗阿尔兹海默症以及其他活性等，具体所涉及的靶点如图3所示。例如，降糖活性涉及的超滤靶点包括：*α*-葡萄糖苷酶（*α*-glucosidase， *α*-Glu）、*α*-淀粉酶（*α*-amylase， *α*-Amy）和蛋白酪氨酸磷酸酶1B（protein tyrosine phosphatase-1B， PTP1B）等。

**图3:**
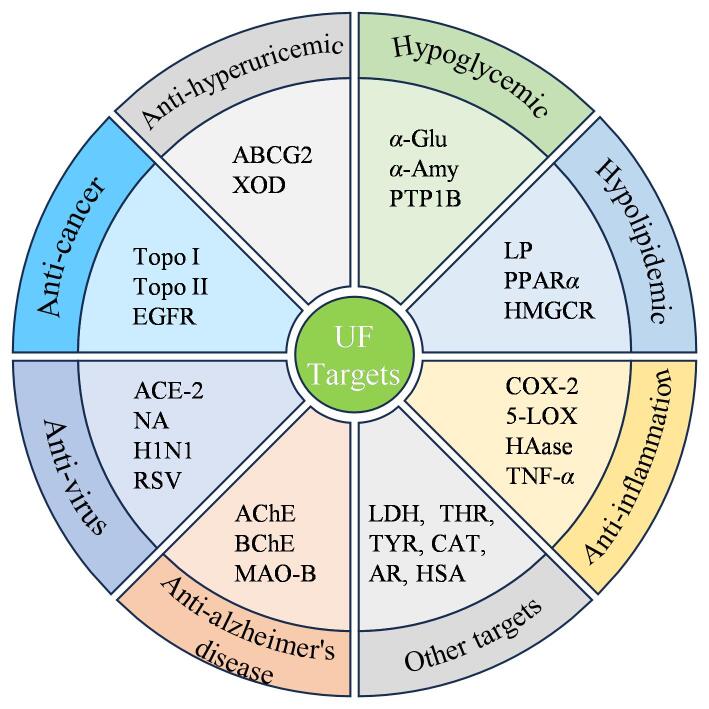
近10年亲和超滤色谱-质谱文章中靶点分类图

进一步对这312篇亲和超滤相关文献中所应用的靶点数量进行统计和分类，如图4a所示。由图4a可知，采用单一靶点进行的超滤筛选文章占比最多，高达78.7%；其次为双靶点的超滤筛选文章，占比为14.08%；而采用3个及以上的多靶点超滤筛选文章数量最少，占比仅为7.22%。另外对这312篇亲和超滤相关文献中报道的研究对象比如中药单味药或复方占比进行统计和分类，如图4b所示。由图4b可知，单方（含化合物水平筛选）超滤筛选的文章占比最多，高达82.05%，综述相关的超滤筛选文章占比次高，为11.22%，而中药复方超滤筛选的文章占比最少，仅为6.73%，其中中药复方超滤筛选的文章近半数为近2年发表，表明近年来利用亲和超滤筛选方法来解析中药复方中的药效物质正成为新的有力手段。

**图4:**
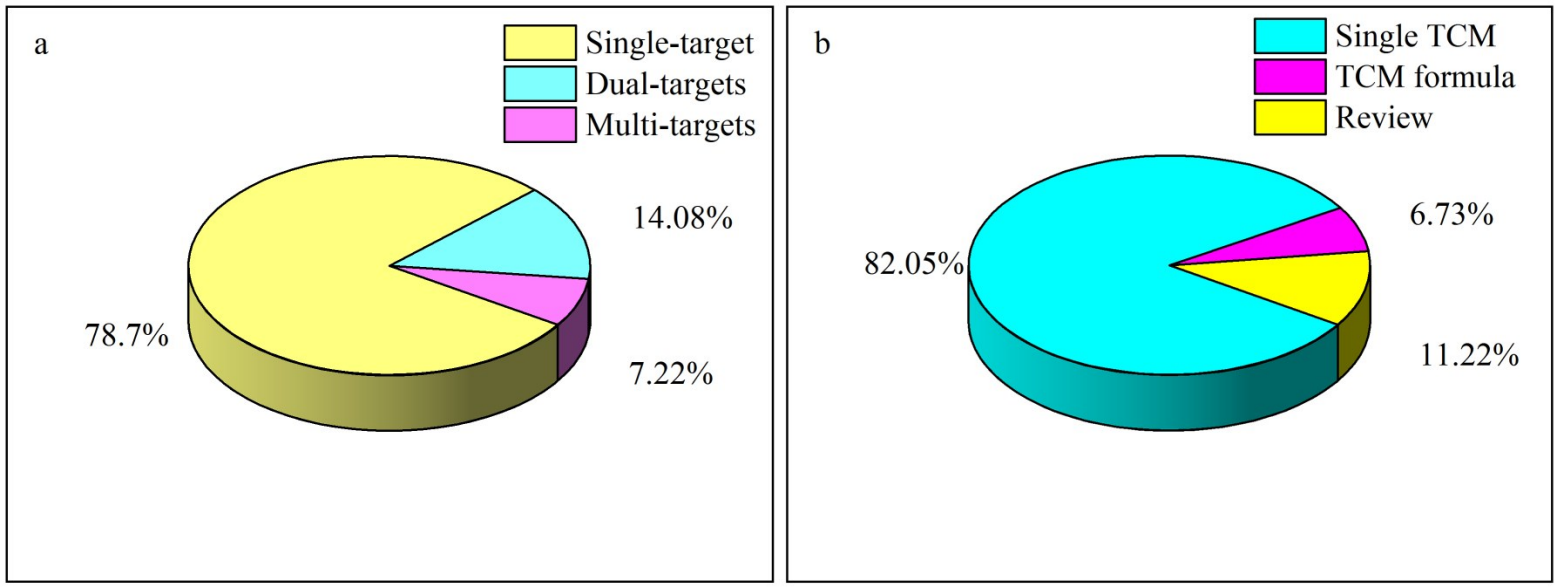
近10年亲和超滤色谱-质谱文章中（a）靶点占比图和（b）中药单复方占比图

## 2 AUF-LC-MS在天然活性成分筛选研究中的应用

### 2.1 AUF-LC-MS在化合物水平的筛选

AUF-LC-MS技术广泛应用于复杂天然产物活性物质的筛选中，也适用于组合化合物库中活性物质的筛选。例如，Zhao等^［9］^将自制的含芒果苷、新芒果苷和四氢小檗碱等10个化合物的混合溶液与环加氧酶2（prostaglandin-endoperoxide synthase 2， PTGS2）靶点进行孵育，利用AUF-LC-MS技术筛选其中潜在的活性物质，结果从10个化合物的混合溶液中筛选出7种化合物与PTGS2靶点具有不同的结合能力，其中新芒果苷的特异性结合值最高，为35.23%，分子对接的验证结果表明新芒果苷与PTGS2靶点的结合能最低，为-42.7 kJ/mol，细胞热转移实验（CETSA）也证实新芒果苷与PTGS2靶点蛋白具有很强的结合亲和力，并进一步通过细胞和斑马鱼试验对新芒果苷抗抑郁的作用机制进行了探究，结果发现新芒果苷可以通过调控PTGS2/EP2/cAMP/Epac1信号通路来发挥抗抑郁的作用。另外，曹娟等^［10］^以唾液酸免疫球蛋白凝集素1（sialic acid binding Ig-like lectin 1， Siglec-1）为靶点，将含有迷迭香酸、阿魏酸、绿原酸、菊苣酸、甘草酸和甘草次酸这6种化合物的混合溶液与Siglec-1靶点进行孵育，利用AUF-LC-MS技术对其进行超滤筛选，结果发现这6种化合物中的甘草次酸与Siglec-1靶点具有较高的特异结合率。其他部分代表性的化合物水平的亲和超滤文献如表1所示。

**表1:** AUF-LC-MS在化合物水平的筛选

Number of compounds	Activity/disease	Target	Active compound	Main verification methods	Ref.
10	depression	PTGS2	neomangiferin	zebrafish， cell， MD	［^9^］
6	immunity	Siglec-1	glycyrrhetinic acid	/	［^10^］
22	anti-tumor	PD-L1	*β*-NAD	cell， MD	［^11^］
6	antimalarial	HSA	piperaquine	MD	［^12^］
5	anti-cancer	Tim-3	atractylenolide I	/	［^13^］

PTGS2： prostaglandin-endoperoxide synthase 2； Siglec-1： sialic acid binding Ig-like lectin 1； PD-L1： programmed cell death ligand 1； HSA： human serum albumin； Tim-3： T cell immunoglobulin and mucin domain-containing protein 3； *β*-NAD： *β*-nicotinamide adenine dinucleotide； MD： molecular docking.

### 2.2 AUF-LC-MS在中药单方水平的筛选

#### 2.2.1 单方水平的单靶标AUF-LC-MS研究

单靶标AUF-LC-MS筛选研究所涵盖的生物活性范围广泛，涉及多种活性相关靶点。例如，抗炎活性所涉及的靶点包括环氧合酶2（cyclooxygenase-2， COX-2）、5-脂氧合酶（5-lipoxygenase， 5-LOX）和肿瘤坏死因子-*α*（tumor necrosis factor-alpha， TNF-*α*）等；降糖活性所涉及的靶点包括*α*-Glu、*α*-Amy和PTP1B等；降脂活性所涉及的靶点包括脂肪酶（lipase， LP）、3-羟基-3-甲基戊二酰辅酶A还原酶（3-hydroxy-3-methylglutaryl Coenzyme A reductase， HMGCR）等；降尿酸活性所涉及的靶点包括黄嘌呤氧化酶（xanthine oxidase， XOD）、ATP结合盒转运蛋白G2（ATP binding cassette transporter G2， ABCG2）等；抗病毒活性所涉及的靶点包括血管紧张素转化酶2（angiotensin-converting enzyme 2， ACE2）、二肽基肽酶 4（dipeptidyl peptidase-4， DPP-4）、甲型H1N1流感病毒（influenza A virus subtype H1N1， H1N1）、呼吸道合胞病毒（respiratory syncytial virus， RSV）等；抗肿瘤活性所涉及的靶点包括DNA拓扑异构酶I（DNA topoisomerase I， Topo I）、DNA拓扑异构酶Ⅱ（DNA topoisomerase Ⅱ， Topo Ⅱ）、表皮生长因子受体（epidermal growth factor receptor， EGFR）等；其他还有美白活性涉及的靶点酪氨酸酶（tyrosinase， TYR）、抗凝血活性涉及的靶点凝血酶（thrombin， THR）、抗糖尿病并发症涉及的靶点醛糖还原酶（aldose reductase， AR）和抗脑卒中涉及的靶点乳酸脱氢酶（lactate dehydrogenase， LDH）等。

Liu等^［14］^以*α*-Glu为靶点，利用AUF-LC-MS技术从土沉香叶中筛选出15种潜在的降糖活性物质，并对这些潜在活性物质进行了细胞试验、酶活试验、分子对接和分子动力学模拟验证，其中表儿茶素、表没食子儿茶素没食子酸酯和二氢杨梅素等5种化合物表现出良好的降糖活性。本研究团队的Chen等^［15］^以COX-2为靶点，利用AUF-LC-MS技术从辣木叶中筛选出15种与COX-2靶点具有不同亲和力的潜在抗炎活性物质，然后对这些潜在活性物质进行构效关系分析，结果表明辣木叶中具有乙酰化糖基侧链的槲皮素和山柰酚黄酮苷是其潜在的抗炎活性成分，并进一步通过分子对接进行了验证。另外，本研究团队的Chen等^［16］^还以Topo I为靶点，利用AUF-LC-MS技术从石蒜中筛选出11种潜在的体外抗增殖活性物质，其中小星蒜碱具有良好的体外抗增殖活性并通过细胞试验对其活性进行了验证。Tian等^［17］^以内皮型一氧化氮合酶（endothelial nitric oxide synthase， eNOS）为靶点，利用AUF-LC-MS-GNPS策略从刺五加中筛选分离得到9种潜在的抗血管内皮损伤活性单体，包括咖啡酸乙酯、刺五加苷B、香草醛、异绿原酸B和芹菜素等，并通过细胞和斑马鱼试验验证了这些活性物质具有改善血管内皮损伤的活性。为了从杜仲茶中筛选出潜在的降脂活性成分，Huang等^［18］^以LP为靶点，利用AUF-LC-MS技术对杜仲茶中的降脂活性成分进行筛选和鉴定，结果成功地从杜仲茶中筛选出15种潜在降脂成分，进一步对其中4个代表性的活性成分（京尼平苷酸、槲皮素-3-*O*-桑布双糖苷、异绿原酸A和槲皮素）进行了体外活性验证，结果证实这4种物质均具有良好的降脂活性。其他单靶点的AUF-LC-MS相关研究信息见表2。

**表2:** AUF-LC-MS在单方水平的筛选

Name of herb	Activity/disease	Targets	Main active compounds	Verification methods	Ref.
*Morus alba* L.	anti-inflammatory	COX-2， 5-LOX	neochlorogenic acid， rutin	cell， EAT， MD	［^19^］
*Nauclea officinalis*	anti-inflammatory	COX-2， 5-LOX	angustine， angustidine	EAT， MD	［^30^］
*Dendropanax dentiger*	anti-inflammatory	COX-2	cryptochlorogenic acid	EAT， MD， MDS	［^31^］
*Curcuma longa*	anti-inflammatory	COX-2	BHPHO	zebrafish， MD	［^32^］
*Moringa oleifera*	anti-inflammatory	COX-2	quercetin 3-acetyl-glycoside	MD	［^15^］
*Aquilaria sinensis*	hypoglycemic	*α*-Glu	epicatechin， epigallocatechin gallate	cell， EAT， MD	［^14^］
Phyllanthi Fructus	hypoglycemic	*α*-Glu	hyperoside， gallic acid， corilagin	EAT， MD	［^33^］
*Diospyros kaki*	hypoglycemic	*α*-Amy， *α*-Glu	hederagenin， ursolic acid	EAT， MD	［^34^］
*Rubus corchorifolius*	hypoglycemic	*α*-Amy， *α*-Glu	cyanidin-3-rutinoside	EAT， MD	［^35^］
*Clematis tangutica*	hypolipidemic	LP	tanguticoside B， rutin	EAT， MD	［^36^］
*Rheum palmatum*	hypolipidemic	LP	procyanidin B5 3，3′-di-*O*-gallate-di-*O*-gallate	EAT， MD	［^37^］
*Polygonum cuspidatum*	anti-hyperuricemic	XOD	polydatin， resveratrol	EAT， MD	［^38^］
*Polyporus umbellatus*	anti-hyperuricemic	XOD	polyporusterone A	EAT， MD， MDS	［^39^］
Cortex Fraxini	anti-hyperuricemic	ABCG2	fraxin	MD	［^40^］
*Bletilla striata*	skin-whitening	TYR	coelonin	zebrafish， MD	［^41^］
*Ampelopsis grossedentata*	skin-whitening	TYR	dihydromyricetin	cell， MD	［^42^］
Semen Oroxyli	skin-whitening	TYR	oroxin A， baicalein	EAT， MD	［^43^］
*Gastrodia elata*	skin-whitening	TYR	4，4′-dihydroxybenzylsulfid	EAT	［^44^］
Peach Kernel	anticoagulant	THR	L-arginine， cytidine	zebrafish， MD	［^45^］
*Salvia miltiorrhiza*	anticoagulant	THR	salvianolic acid C	EAT， MD	［^46^］
*Ligusticum chuanxiong*	anticoagulant	THR	ferulic acid， chlorogenic acid	EAT， MD	［^47^］
*Oenothera biennis*	diabetes complications	AR	gallic acid， catechin	EAT	［^48^］
*Caesalpinia spinose*	diabetes complications	AR	methyl gallate， pistafolin B	EAT， MD	［^49^］
*Polygala tenuifolia*	anti-stroke	LDH	sibiricose A5， glomeratose A	cell	［^50^］
*Glycine max* L.	anti-stroke	LDH	daidzin， glycitin， genistin	cell	［^51^］
Bupleuri Radix	anti-influenza virus	NA	6″-*O*-acetylsaikosaponin A	EAT， MD	［^52^］
*Polygonum cuspidatum*	anti-influenza virus	NA	*cis*-polydatin	EAT， MD	［^53^］
*Dalbergia odorifera*	alzheimer’s disease	AChE	tectorigenin， fisetin， dalbergin	EAT， MD	［^54^］
*Astragalus membranaceus*	alzheimer’s disease	AChE， 5-LOX	calycosin-7-glucoside	MD	［^55^］
*Radix Paeoniae alba*	anti-allergy	HDC	catechin	EAT， MD	［^56^］
*Glycyrrhiza uralensis*	antiviral	H1N1， RSV	glycyrrhizic acid， licochalcone A	cell	［^57^］
*Scutellaria baicalensis*	antiviral	ACE2， DPP-4	wogonoside， chrysin， oroxylin A	/	［^58^］
*Moringa oleifera*	anti-aging	HAase， Ela， Col	4-caffeoylquinic acid， vicenin-2	MD	［^59^］
*Rodgersia podophylla*	antioxidant， anti-hyperuricemic	SOD， XOD	norbergenin， bergenin， catechin	EAT， MD	［^21^］
*Polygonatum sibiricum*	antioxidant， anti-hyperuricemic	SOD， XOD	*N*-*cis*-*p*-coumaroyltyramine	MD	［^60^］
*Moringa oleifera*	hypoglycemic， hypolipidemic	*α*-Glu， LP	kaempferol 3-acetyl-glycoside	MD	［^61^］
*Cyclocarya paliurus*	hypoglycemic， hypolipidemic	*α*-Glu， LP	chlorogenic acid， quercetin	MD	［^62^］
*Xylopia aethiopica*	hypoglycemic， hypolipidemic	*α*-Glu， LP	liriodenine， oxonantenine	cell， EAT	［^63^］
*Morus alba* L.	hypoglycemic， hypolipidemic	*α*-Glu， LP	chlorogenic acid	EAT， MD	［^64^］
*Azadirachta indica*	antiparasitic	AChE， LDH	carnosol	EAT， MD	［^22^］
*Hagenia abyssinica*	antiparasitic	AChE， LDH， GR	corilagin， brevifolin， quercetin	EAT， MD	［^65^］
*Andrographis paniculata*	anti-inflammatory， antiviral	COX-2， IL-6， ACE2	andrographolide	MD	［^66^］
*Rhamnus davurica*	anti-inflammatory， anti-tumor	COX-2， Topo I	vitexin， apigenin， kaempferol	EAT， cell	［^67^］

COX-2： cyclooxygenase-2； 5-LOX： 5-lipoxygenase； *α*-Glu： *α*-glucosidase； *α*-Amy： *α*-amylase； LP： lipase； XOD： xanthine oxidase； ABCG2： ATP binding cassette transporter G2； TYR： tyrosinase； THR： thrombin； AR： aldose reductase； LDH： lactate dehydrogenase； NA： neuraminidase； AChE： acetylcholinesterase； HDC： histidine decarboxylase； H1N1： influenza A virus subtype H1N1； RSV： respiratory syncytial virus； ACE2： angiotensin-converting enzyme 2； DPP-4： dipeptidyl peptidase-4； HAase： hyaluronidase； Ela： elastase； Col： collagenase； SOD： superoxide dismutase； GR： glutathione reductase； IL-6： interleukin-6； Topo I： DNA topoisomerase I； BHPHO： （*E*）-1，7-bis（4-hydroxyphenyl）-6-hepten-3-one； EAT： enzyme activity test； MDS： molecular dynamics simulations.

#### 2.2.2 单方水平的双靶标AUF-LC-MS研究

近年来，利用AUF-LC-MS进行双靶标超滤筛选的研究越来越多。例如，Du等^［19］^以5-LOX和COX-2为双靶点，利用AUF-LC-MS技术从桑叶中共筛选出17种潜在的活性物质，进一步通过分子对接、体外酶活和细胞试验对这些筛选出来的活性物质进行验证，结果发现新绿原酸、芦丁、异槲皮素和（-）-丁香树脂-4-*O*-葡萄糖苷可能是桑叶中调节肥胖和2型糖尿病花生四烯酸代谢的潜在物质基础。Liu等^［20］^以5-LOX和乙酰胆碱酯酶（acetylcholinesterase， AChE）为双靶点，利用AUF-LC-MS技术从黄芪花中共筛选出7种潜在的抗炎和抗神经性疾病活性物质，其中9，10-二甲氧基-菜豆素-3-*O*-*β*-D-葡萄糖苷对5-LOX和AChE靶标具有强效的双重抑制作用，其IC_50_值分别为5.69×10^-5^ mol/L和3.97×10^-5^ mol/L。本研究团队的Liang等^［21］^以超氧化物歧化酶（superoxide dismutase， SOD）和XOD为双靶点，利用AUF-LC-MS技术从鬼灯檠中共筛选出6种潜在的抗氧化和降尿酸活性配体，并对这些潜在的活性配体进行了分子对接和体外酶活试验的验证，结果发现岩白菜素和儿茶素是鬼灯檠中主要的降尿酸活性成分。另外，本研究团队的Fan等^［22］^以AChE和LDH为双靶点，利用AUF-LC-MS技术从印楝中共筛选出14种潜在的抗寄生虫活性物质，并对这些潜在的活性成分进行了分子对接和体外酶活试验的验证，结果发现鼠尾草酚为印楝中潜在的强效AChE和LDH双重抑制剂，其中鼠尾草酚对AChE靶点的IC_50_值为（40.66±3.10） μmol/L。为了筛选山苦茶中潜在的降糖活性物质，Li等^［23］^以*α*-Glu和*α*-Amy为双靶点，利用AUF-LC-MS技术对其潜在降糖物质进行筛选和鉴定，结果从中筛选并鉴定出10种活性成分，经过体外酶活及分子对接验证发现山苦茶中的儿茶素、表儿茶素、芦丁、阿魏酸和山奈苷是其主要的降糖活性物质。上述这些AUF-LC-MS研究中采用的双靶点既有属于相同活性的，也有属于两种不同的生物活性的。

#### 2.2.3 单方水平的多靶标AUF-LC-MS研究

近几年来，多靶标筛选已逐渐成为AUF-LC-MS研究的热点。例如，Hou等^［24］^以LDH、脂肪氧合酶（lipoxygenase， LOX）和神经氨酸酶（neuraminidase， NA）为多靶点，利用AUF-LC-MS技术结合逆流色谱方法从紫花苜蓿中共筛选分离得到3种潜在的活性物质，其中槲皮素、染料木素和芒柄花黄素这3种化合物分别被鉴定为强效的NA、LOX和LDH抑制剂。为了筛选人参中潜在的神经保护物质，Yu等^［25］^利用AChE、B型单胺氧化酶（monoamine oxidase B， MAO-B）和*N*-甲基天冬氨酸（*N*-methyl-D-aspartic acid， NMDA）受体为多靶点进行AUF-LC-MS筛选，结果成功地从中筛选出16种潜在的神经保护活性物质，并对其中部分代表性活性物质进行了体外酶活及分子对接验证。本研究团队的Xu等^［26］^以*α*-Glu、*α*-Amy、LP和XOD为多靶点，利用AUF-LC-MS技术从大黄中共筛选出26种潜在的降糖、降脂和降尿酸活性物质，并进一步对部分代表性活性物质进行了分子对接和体外酶活验证，结果发现表儿茶素-3-*O*-没食子酸酯是大黄中潜在的降糖、降脂和降尿酸活性成分，另外芦荟大黄素-8-*O*-葡萄糖苷、大黄素-8-*O*-葡萄糖苷和大黄酸等也在大黄的降糖、降脂和降尿酸活性中发挥重要的贡献作用，据此也是首次构建了大黄中潜在降糖、降脂和降尿酸的多成分-多靶点作用网络图，其研究示意图如图5所示。另外，本研究团队的Xu等^［27］^还以*α*-Glu、*α*-Amy、PTP1B、LP和HMGCR为多靶点，利用AUF-LC-MS技术从黄芩中共筛选出20种潜在的降糖、降脂活性物质，并进一步对部分代表性活性物质进行了分子对接和体外酶活验证，结果发现黄芩中的黄芩素、汉黄芩素和汉黄芩苷具有显著的降血糖和降血脂作用。本研究团队的Zhang等^［28］^以*α*-Glu、LP和COX-2为多靶点，利用AUF-LC-MS技术从荷叶中共筛选出11种潜在的降糖、降脂和抗炎活性物质，并进一步对部分代表性活性物质进行了分子对接和体外酶活验证，结果发现*N*-去甲基荷叶碱、荷叶碱、2-羟基-1-甲氧基阿朴啡和异鼠李素-3-*O*-葡萄糖苷是荷叶中潜在的降糖、降脂和抗炎活性成分。另外，本研究团队的Feng等^［29］^以Topo I、Topo Ⅱ、COX-2和ACE2为多靶点，利用AUF-LC-MS技术从八角莲中共筛选出12种潜在的抗肿瘤、抗炎和抗病毒活性物质，并进一步对部分代表性活性物质进行了分子对接、体外酶活和细胞试验验证，结果发现八角莲中的鬼臼毒素和槲皮素对肺癌A549和结直肠癌HT-29细胞的抑制率与阳性药5-氟尿嘧啶（fluorouracil， 5-FU）和依托泊苷相当，山柰酚对COX-2具有很强的抑制作用且其IC_50_值为（0.36±0.02） μmol/L，优于阳性药吲哚美辛的（0.73±0.07） μmol/L，另外槲皮素对ACE2也具有很强的抑制作用，其IC_50_值为（104.79±8.26） μmol/L，上述结果表明八角莲中的鬼臼毒素、山柰酚和槲皮素是其发挥抗肿瘤、抗炎和抗病毒的药效物质基础。越来越多的多靶标AUF-LC-MS研究应用于中药药效物质的筛选当中，为中药往往通过多成分-多靶点-多途径发挥疗效提供了重要的科学依据。其他多靶点的AUF-LC-MS相关研究信息见表2。

**图5:**
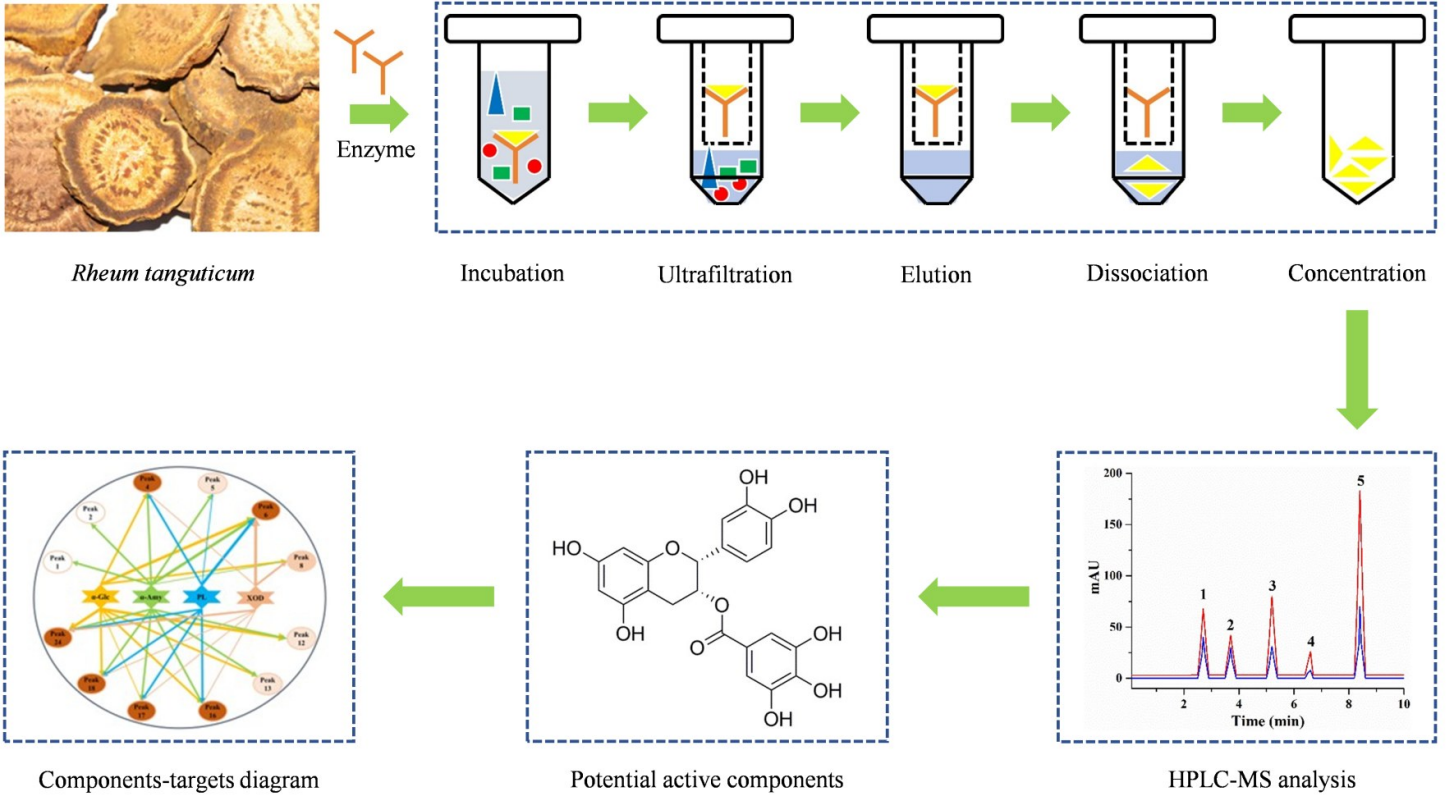
大黄降糖、降脂和降高尿酸活性成分筛选的AUF-LC-MS研究示意图

### 2.3 AUF-LC-MS在中药复方水平的筛选

近年来，AUF-LC-MS技术在中药复方的药效物质基础筛选中也得到了广泛应用。例如，Liu等^［68］^以脯氨酸羟化酶2（human proline hydroxylase 2， PHD2）和受体相互作用蛋白激酶3（receptor interacting serine threonine kinase 3， RIPK3）为靶点，利用AUF-LC-MS技术从桃核承气汤复方中共筛选出12种潜在的活性物质，并进一步对部分代表性活性物质进行了细胞试验、表面等离子共振（surface plasmon resonance， SPR）、细胞热转移分析（cellular thermal shift assay， CETSA）和药物亲和力反应靶标稳定性（drug affinity responsive target stability， DARTS）验证，结果发现桃核承气汤复方中的苦杏仁苷和大黄酸是通过调控PHD2/UP1和RIPK3/AKT/TGF-*β*信号通路来发挥缓解慢性肾功能衰竭的主要药效物质。Huang等^［69］^以THR为靶点，利用AUF-LC-MS技术从血府逐瘀汤复方中筛选出8种潜在的抗凝血成分，包括氧化芍药苷、绿原酸、苦杏仁苷、异甘草苷、新橙皮苷、橙皮苷、甘草酸和竹节参皂苷Ⅳa，除已报道的具有良好的抗凝血作用的竹节参皂苷Ⅳa外，作者进一步对另外7种活性物质进行了分子对接、体外凝血因子试验、体外酶活和斑马鱼试验验证，结果表明这7种活性成分也具有良好的抗凝血作用，未来可以作为新的抗血栓药物进行进一步的研究和开发。另外，Fu等^［70］^首先通过网络药理学分析获得芍药甘草附子汤复方治疗类风湿性关节炎的靶点为TNF-*α*，再以TNF-*α*为靶点结合AUF-LC-MS技术从芍药甘草附子汤复方中筛选出6种潜在的抗炎活性成分，包括甘草酸、芍药苷、芒柄花黄素、异甘草素、苯甲酰新乌头原碱和甘草次酸，并进一步对这些活性成分进行了分子对接、分子动力学和细胞试验验证，结果证实这些活性成分均具有良好的体外抗炎活性，表明这些活性成分是芍药甘草附子汤复方中潜在的抗炎药效物质基础。本研究团队的Xu等^［71］^以*α*-Glu、*α*-Amy和LP为靶点，利用AUF-LC-MS技术从三黄汤复方中共筛选出28种潜在的降糖、降脂活性物质，并进一步对部分代表性活性物质进行了分子对接和体外酶活验证，结果发现三黄汤复方中的表儿茶素-3-*O*-没食子酸酯具有显著的降糖降脂作用。上述研究结果表明AUF-LC-MS是一种可应用于复杂中药复方药效物质快速、高效筛选的技术，为其他中药复方药效物质的快速筛选提供了一个重要参考。另外，其他中药复方相关的AUF-LC-MS研究信息见表3。

**表3:** AUF-LC-MS在复方水平的筛选

Name of compound recipe	Activity/disease	Targets	Main active compounds	Main verification methods	Ref.
TaoHeChengQi decoction	chronic renal failure	PHD2， RIPK3	amygdalin， rhein	cell， SPR， CETSA	［^68^］
Xuefu Zhuyu decoction	anticoagulant	THR	amygdalin， isoliquiritin	zebrafish， MD	［^69^］
Shaoyao Gancao Fuzi decoction	rheumatoid arthritis	TNF-*α*	paeoniflorin， formononetin	cell， MD， MDS	［^70^］
Sanhuang decoction	T2DM， obesity	*α*-Glu， *α*-Amy， PL	epicatechin-3-*O*-gallate	EAT， MD	［^71^］
Zuogui pill	cancer bone metastasis	RANKL	luteolin	MD， CETSA	［^72^］
TongFengTangSan	hyperuricemia	XOD	1，3，6-trigalloylglucose	cell， EAT， MD	［^73^］
Fufang Qiling granules	hyperuricemia	XOD	taxifolin， epicatechin， ferulic acid	SPR， MD	［^74^］
Shenqi Jiangtang granule	diabetes complications	AR	ginsenoside Rd， gomisin J	EAT， MD	［^75^］
Danggui-shaoyao-san	nephrotic syndrome	uPA， PLA	1，2，3，4，6-*O*-pentagalloylglucose	EAT， MD	［^76^］
Zi-shen pill	anti-inflammatory	5-LOX	anemarrhenasaponin I	EAT， MD	［^77^］

T2DM： type 2 diabetes mellitus； PHD2： human proline hydroxylase 2； RIPK3： receptor interacting serine threonine kinase 3； TNF-*α*： tumor necrosis factor-alpha； RANKL： tumor necrosis factor ligand superfamily member 11； XOD： xanthine oxidase； uPA： urokinase-type plasminogen activator； PLA： plasmin； SPR： surface plasmon resonance； CETSA： cellular thermal shift assay.

## 3 天然活性物质筛选其他方法进展

目前，已有多个研究团队开发多种筛选天然活性成分的方法，如传统的植化分离后筛选、细胞膜色谱、毛细管电泳、类器官、谱-效关系、分子对接和网络药理学等。例如，Zhao等^［78］^利用传统的植化提取分离技术从泰国的木果楝种子中分离得到9种新的柠檬苦素类化合物和2种新的原柠檬苦素类化合物，其中化合物Thaigranatum G具有显著的神经保护活性，包括降低BV2小鼠小胶质细胞促炎因子的表达如COX2、iNOS和IL-1*β*，以及对小鼠中脑多巴胺能神经元MN9D细胞也表现出神经保护作用。Liu等^［79］^通过细胞膜色谱技术从五味子中筛选得到8种潜在的抗肝纤维化活性物质，其中Gomisin N具有显著的抗肝纤维化活性，其潜在的机制可能与减少炎症反应、减轻氧化应激、抑制肝微血管生成和减轻肝细胞凋亡有关。Yan等^［80］^建立了一种高效、环保的毛细管电泳在线筛选脂肪酶抑制剂的方法，并从橘皮中筛选出桔皮素、川陈皮素、橙皮素和柚皮素等多个潜在的脂肪酶抑制剂。王培等^［81］^构建了人源结直肠腺瘤（colorectal adenoma， CRA）复发类器官模型并利用该类器官模型评价了7种中药活性成分，结果发现这7种中药活性成分中的大黄素和黄芩素具有治疗 CRA 复发的潜力。Li等^［82］^利用谱-效关系分析结合化学计量学手段从36批荷叶样品中筛选出槲皮素3-*O*-葡萄糖酸苷为荷叶中潜在的降脂活性成分，并通过体外酶活试验、细胞试验和斑马鱼试验对其进行活性验证。Pantaleão等^［83］^为了筛选出潜在的降糖活性先导化合物，以DPP-4为靶点，利用分子对接技术开展虚拟筛选，结果从1 880个化合物库中筛选出1个潜在的候选化合物（ZINC1572309），并发现该化合物在非细胞毒性浓度下具有良好的DPP-4抑制活性。张喆等^［84］^利用网络药理学手段从活络效灵丹中筛选出3-乙酰基-11-酮基-*β*-乳香酸、11-酮基-*β*-乳香酸、丹参酮ⅡB、洋川芎内酯F、阿魏酸等5个潜在的治疗糖尿病周围神经病变的药效物质基础。本文对这些不同的天然活性物质筛选方法的优缺点进行比较分析（表4），这有助于不同研究者根据不同研究对象和研究目标选择最适合的筛选方法。例如，虽然传统的植化分离耗时长、成本高，但是可以直接获得活性单体，对于后期的活性验证可以直接提供样本。而细胞膜色谱虽然具有高通量和高选择性等优点，但是也存在假阳性、操作复杂等缺点，需要通过后期的验证从而排除假阳性的结果，另外，与AUF-LC-MS技术相比，细胞膜色谱虽然同样具有高通量和高选择性等优点，但是由于其操作较为复杂，相较于AUF-LC-MS技术，细胞膜色谱方法的可推广性弱一些，其他的一些优缺点对比如表4所示。

**表4:** 不同天然活性成分筛选方法的比较

Screening methods	Advantages	Disadvantages
Traditional phytochemical separation methods	obtaining active compounds and verifying their activities	high cost， low efficiency
Cell membrane chromatography	high-throughput， high sensitivity， automation	false positive cases， complex operation
Capillary electrophoresis	high separation efficiency， fast analysis speed， simple operation， and low sample and reagent consumption	complex operation， insufficient popularity
Organoids methods	no ethical issues， approximating real human conditions	no standardized organoid construction system
Spectrum-effect relationship	reflecting the overall efficacy of traditional Chinese medicine and the impact of component changes on efficacy	statistical methods dependency
Molecular docking	low cost， fast calculation speed， flexibility	false positive cases
Network pharmacology	low cost， integrating multi-omics data	database dependency

## 4 天然活性物质筛选及AUF-LC-MS研究展望

天然药物中的化学成分众多且十分复杂，如何快速筛选并阐释其中的生物活性物质一直是其药效物质基础研究以及新药研发的难题。传统的植化提取、分离获取单体成分再进行活性评价往往是费时费力且成本高昂，AUF-LC-MS则具有所需样品少、特异性高、灵敏度高、快速高效、操作简单且可以高通量筛选等诸多优点。其中，相较于传统的单靶点AUF-LC-MS筛选，多靶点AUF-LC-MS筛选近年来也是得到了研究者们的广泛应用。由于中药尤其是中药复方往往是通过多成分-多靶点-多途径的效应来改善相应疾病，因此多靶点AUF-LC-MS筛选更贴合中药及中药复方通过整体观来发挥功效的作用。然而，由于目前多靶点AUF-LC-MS筛选研究仍处于初期阶段，也存在着一些问题。例如，多数的多靶点AUF-LC-MS筛选研究中应用的多靶点仍是独立的单靶点筛选的叠加，而不是多个靶点的混合筛选，未来可考虑将多个靶点混合一块再对中药或中药复方进行多靶点筛选，从而更贴近多靶点筛选的内涵。

另外，AUF-LC-MS也存在一些局限性，主要有以下几点。一是小分子物质与超滤膜或者靶点蛋白非活性位点的非特异性吸附会导致出现假阳性结果，解决办法是首先采用失活蛋白作为阴性对照以排除假阳性结果；其次还可以通过体外酶活试验及细胞试验等活性验证进一步排除假阳性结果，从而提高AUF-LC-MS的准确度及有效性。二是当前应用AUF-LC-MS技术进行活性成分筛选的研究多集中于单一靶点，而针对双靶点或多靶点的筛选研究仍较为有限，尤其是在中药复方领域的多靶点筛选研究仍处于初步发展阶段，未来可结合多靶点筛选复杂天然药物如中药复方中的潜在药效物质，这也更贴合中药往往通过多成分-多靶点-多途径发挥功效的整体观特点。三是当前利用AUF-LC-MS进行活性成分筛选后得到的活性物质多数仅进行分子对接和体外酶活实验验证，而基于类器官、细胞和动物水平的验证仍较少，未来对于筛选出的活性物质可以进行更多维度的活性验证。而且，对于活性显著的物质还可以进一步对其结构进行优化，从而为新药研发提供良好的基础。四是当前AUF-LC-MS筛选研究大多集中于传统的一些降糖、降脂和抗炎等活性的靶点，对于一些新颖生物活性对应的新作用靶点研究仍较少，未来可结合人工智能和机器学习算法等方法获取一些新的活性作用靶点，并将筛选所得的活性物质、作用靶点、治疗疾病及药物类别等信息整合至同一个智能化数据平台，从中药整体化特色阐释其药效物质基础，从而进一步推动中药的现代化发展。

随着科学技术的发展，除AUF-LC-MS外，未来天然活性物质筛选还可以结合类器官、生物芯片和人工智能等新兴技术进行筛选研究，同时还可以应用多种不同的筛选方法进行多维度组合筛选，进一步拓展其应用范围并增加其精准度和通量。

## References

[1] Bernardini S， Tiezzi A， Laghezza Masci V， et al . Nat Prod Res， 2018， 32（16）： 1926 28748726 10.1080/14786419.2017.1356838

[2] Newman D J， Cragg G M . J Nat Prod， 2020， 83（3）： 770 32162523 10.1021/acs.jnatprod.9b01285

[3] Hou X F， Sun M， Bao T， et al . Acta Pharm Sin B， 2020， 10（10）： 1800 33163336 10.1016/j.apsb.2020.04.016 PMC7606101

[4] Jiao X Y， Jin X， Ma Y Y， et al . Comput Biol Chem， 2021， 90： 107402 33338839 10.1016/j.compbiolchem.2020.107402

[5] He Y Q， Zhao X Y， Yu M Z， et al . Molecules， 2025， 30（3）： 608 39942712

[6] Chen G L， Huang B X， Guo M Q . Phytochem Anal， 2018， 29（4）： 375 29785715 10.1002/pca.2769

[7] Zhou H， Wang Y M， Zheng Z， et al . Journal of Chinese Mass Spectrometry Society， 2018， 39（6）： 641

[8] Van Breemen R B， Huang C R， Nikolic D， et al . Anal Chem， 1997， 69（11）： 2159 9183179 10.1021/ac970132j

[9] Zhao D P， Xue A， Yuan K， et al . J Ethnopharmacol， 2025， 347： 119792 40216047 10.1016/j.jep.2025.119792

[10] Cao J， Yang Z J， Zheng Y， et al . Drug Evaluation Research， 2024， 47（7）： 1556

[11] Huang Q Y， Zang X L， Zhang Z W， et al . Int J Biol Macromol， 2023， 230： 123266 36646351 10.1016/j.ijbiomac.2023.123266

[12] Ma R， Guo D X， Li H F， et al . Spectrochimica Acta， Part A， 2019， 222： 117158 31181505 10.1016/j.saa.2019.117158

[13] Yang Z J， Li S S， Cao J， et al . Drug Evaluation Research， 2023， 46（3）： 545

[14] Liu Y X， Li F L， Fei T， et al . Food Chem， 2025， 463： 141329 39305674 10.1016/j.foodchem.2024.141329

[15] Chen G L， Xu Y B， Wu J L， et al . ACS Food Sci Technol， 2021， 1（10）： 1953

[16] Chen G L， Tian Y Q， Wu J L， et al . Sci Rep， 2016， 6（1）： 38284 27922057 10.1038/srep38284 PMC5138836

[17] Tian Z H， Chi B Q， Liu W B， et al . Food Funct， 2025， 16（8）： 3134 40159904 10.1039/d4fo04856c

[18] Huang H， Han M H， Gu Q， et al . Food Chem， 2023， 426： 136630 37352710 10.1016/j.foodchem.2023.136630

[19] Du Y， Chen Y L， Zhang Y， et al . J Ethnopharmacol， 2025， 341： 119325 39761838 10.1016/j.jep.2025.119325

[20] Liu R Y， Zhang Y C， Li S N， et al . Ind Crops Prod， 2025， 225： 120504

[21] Liang C， Xu Y B， Fan M X， et al . Front Pharmacol， 2023， 14： 1298049 38027025 10.3389/fphar.2023.1298049 PMC10663331

[22] Fan M X， Rakotondrabe T F， Chen G L， et al . J Anal Test， 2024， 8（4）： 403

[23] Li S J， Zhang W M， Wang R M， et al . Food Chem， 2022， 388： 133004 35483282 10.1016/j.foodchem.2022.133004

[24] Hou W C， Liu C M， Xia J L， et al . Phytochem Anal， 2021， 32（3）： 382 32893385 10.1002/pca.2985

[25] Yu L D， Wei F T， Liang J， et al . Am J Chin Med， 2019， 47（06）： 1345 31495181

[26] Xu Y B， Chen G L， Guo M Q . Food Front， 2023， 4（2）： 922

[27] Xu Y B， Li Y W， Chen G L， et al . Phytochem Anal， 2024， 35（2）： 239 37779216 10.1002/pca.3285

[28] Zhang H， Chen G L， Zhang Y L， et al . Food Chem， 2022， 375： 131856 34942503 10.1016/j.foodchem.2021.131856

[29] Feng H X， Chen G L， Zhang Y L， et al . J Inflammation Res， 2022， 15： 4677 10.2147/JIR.S371830 PMC9392260 35996684

[30] Wang G H， Tang X Q， Qin F X， et al . Microchem J， 2024， 207： 112171

[31] Wang Q H， Xia B W， Liu R H， et al . Chem Biodiversity， 2025， 22（7）： e202500090 10.1002/cbdv.202500090 40062490

[32] Lan Z W， Yang R， Wang H， et al . Bioorg Chem， 2024， 147： 107357 38604020 10.1016/j.bioorg.2024.107357

[33] Zhang Y F， Yu Q， Tan P， et al . Food Funct， 2025， 16（2）： 422 39564797 10.1039/d4fo04198d

[34] Han Z X， Ren W W， Liu X J， et al . Int J Biol Macromol， 2024， 257： 128616 38070815 10.1016/j.ijbiomac.2023.128616

[35] Li F L， Luo T J， Hou J L， et al . LWT， 2023， 181： 114763

[36] Wei Y F， Chen T， Song H， et al . Phytochem Anal， 2025， 36（1）： 101 39009466 10.1002/pca.3422

[37] Quan S H， Wen M Y， Xu P， et al . Phytochem Anal， 2024， 35（3）： 540 38053479 10.1002/pca.3311

[38] Guo H Y， Hu S M， Ran H Y， et al . J Pharm Biomed Anal， 2024， 243： 116103 38492510 10.1016/j.jpba.2024.116103

[39] Liu Q， Zhuang S Y， Li S N， et al . Phytochem Anal， 2024， 35（1）： 116 37798938 10.1002/pca.3279

[40] Huang X X， Dong W Q， Luo X， et al . Molecules， 2023， 28（23）： 7896 38067624 10.3390/molecules28237896 PMC10708028

[41] Chu C， Li J X， Li C Y， et al . J Chromatogr B， 2023， 1224： 123728 10.1016/j.jchromb.2023.123728 37182408

[42] Zhou H J， Zhang L， Li T Z， et al . Chinese Traditional and Herbal Drugs， 2021， 52（22）： 6796

[43] Yin X S， Zhang X Q， Yin J T， et al . J Chromatogr Sci， 2019， 57（9）： 838 31504273 10.1093/chromsci/bmz054

[44] Wang Z Q， Hwang S H， Lim S S . J Chromatogr A， 2017， 1529： 63 29132823 10.1016/j.chroma.2017.11.008

[45] Huang S Y， Zhang J J， He X C， et al . Chem Biodiversity， 2025， 22（2）： e202401239 10.1002/cbdv.202401239 39327817

[46] Hu H， Lai L， Wang S M， et al . Chinese Traditional and Herbal Drugs， 2024， 55（21）： 7217

[47] Huang C C， Zhao Y， Huang S Y， et al . Phytochem Anal， 2023， 34（4）： 443 37038738 10.1002/pca.3225

[48] Wang Z Q， Shen S G， Cui Z， et al . Molecules， 2019， 24（15）： 2709 31349647 10.3390/molecules24152709 PMC6695788

[49] Wang Z Q， Guillen Quispe Y N， Hwang S H， et al . Ind Crops Prod， 2018， 122： 709

[50] Li S N， Liu S， Liu Z Q， et al . J Sep Sci， 2017， 40（6）： 1385 28134488 10.1002/jssc.201601216

[51] Liu C M， Li S N， Tsao R， et al . J Funct Foods， 2017， 31： 295

[52] Tian Z H， Ma Z， Chi B Q， et al . Chinese Traditional and Herbal Drugs， 2025， 56（3）： 779

[53] Wang L Q， Chen M H， Sun Q H， et al . J Sep Sci， 2023， 46（10）： 2200937 10.1002/jssc.202200937 36905353

[54] Zhang H B， Liu Y C， Zhang L， et al . J Sep Sci， 2024， 47（14）： 2400288 10.1002/jssc.202400288 39034832

[55] Liu R Y， Zhang Y C， Li S N， et al . J Sep Sci， 2023， 46（4）： 2200812 10.1002/jssc.202200812 36502278

[56] Hu G Z， Wang L， Li X Q， et al . J Chromatogr A， 2023， 1693： 463859 36868086 10.1016/j.chroma.2023.463859

[57] Li Z Y， Dong M Y， Chen Z N， et al . J Ethnopharmacol， 2025， 338： 118978 39433166 10.1016/j.jep.2024.118978

[58] Kang J B， Zhang Y C . Journal of Changchun University of Chinese Medicine， 2024， 40（8）： 859

[59] Xu Y B， Chen G L， Guo M Q . Front Nutr， 2022， 9： 854882 35619958 10.3389/fnut.2022.854882 PMC9127542

[60] Li J， Wang Z， Fan M X， et al . Antioxidants， 2022， 11（9）： 1651 36139724

[61] Chen G L， Xu Y B， Wu J L， et al . Food Chem， 2020， 333： 127478 32663752 10.1016/j.foodchem.2020.127478

[62] Xu M J， Feng H X， Guo M Q . eFood， 2024， 5（1）： e123

[63] Liu Y， Li Y W， Muema F W， et al . J Funct Foods， 2023， 106： 105601

[64] Zhang H， Chen G L， Zhang Y L， et al . J Anal Test， 2024， 8（3）： 315

[65] Fan M X， Guo M Q， Chen G L， et al . J Ethnopharmacol， 2024， 332： 118356 38763372 10.1016/j.jep.2024.118356

[66] Feng H X， Chen G L， Guo M Q . Food Chem， 2023， 404： 134515 36240559 10.1016/j.foodchem.2022.134515

[67] Chen G L， Wu J L， Li N， et al . Anal Bioanal Chem， 2018， 410（15）： 3587 29476234 10.1007/s00216-018-0953-6

[68] Liu W， Hu C， Qian X J， et al . Phytomedicine， 2025， 141： 156548 40112631 10.1016/j.phymed.2025.156548

[69] Huang C C， Dai J， He X C， et al . Anal Methods， 2024， 16（44）： 7576 39377216 10.1039/d4ay01162g

[70] Fu W L， Shentu C Y， Chen D， et al . J Ethnopharmacol， 2024， 330： 118268 38677569 10.1016/j.jep.2024.118268

[71] Xu Y B， Guo M Q . Journal of Chinese Mass Spectrometry Society， 2024， 45（6）： 811

[72] Li B H， Chen Z C， Zhang Z Y， et al . Phytomedicine， 2024， 123： 155257 38103318 10.1016/j.phymed.2023.155257

[73] Wu J Y， An Q， Liao Z G， et al . Chinese J Anal Chem， 2025， 53（7）： 100551

[74] Ye J M， Yao J Y， Xu S J， et al . J Ethnopharmacol， 2024， 333： 118410 38848973 10.1016/j.jep.2024.118410

[75] Zhang H， Xu C， Tian Q H， et al . J Ethnopharmacol， 2021， 264： 113282 32890716 10.1016/j.jep.2020.113282

[76] Yang M， Ni L H， Wang Y L， et al . J Ethnopharmacol， 2022， 292： 115171 35259444 10.1016/j.jep.2022.115171

[77] Wang S Q， Huai J X， Shang Y， et al . J Ethnopharmacol， 2020， 254： 112733 32145333 10.1016/j.jep.2020.112733

[78] Zhao W， Luo F L， Liu L F， et al . J Nat Prod， 2025， 88（4）： 1030 40228158 10.1021/acs.jnatprod.5c00137

[79] Liu F B， Li S N， Gu Y Q， et al . J Ethnopharmacol， 2025， 351： 120067 40484259 10.1016/j.jep.2025.120067

[80] Yan T C， Yue Z X， Gu Y X， et al . J Food Compos Anal， 2022， 105： 104185

[81] Wang P， Xia W T， Zhang Z， et al . Drug Evaluation Research， 2025， 48（8）： 2109

[82] Li J Y， Jiang Z M， Wang J， et al . J Pharm Biomed Anal， 2024， 249： 116337 38986347 10.1016/j.jpba.2024.116337

[83] Pantaleão S Q， Philot E A， De Paula H， et al . Biochimie， 2022， 194： 43 34952193 10.1016/j.biochi.2021.12.007

[84] Zhang Z， Li Y Q， Shi Y N， et al . Acta Pharmaceutica Sinica， 2025， 60（4）： 1124

